# Assessing the impact of a restrictive opioid prescribing law in West Virginia

**DOI:** 10.1186/s13011-021-00349-y

**Published:** 2021-02-01

**Authors:** Cara L. Sedney, Maryam Khodaverdi, Robin Pollini, Patricia Dekeseredy, Nathan Wood, Treah Haggerty

**Affiliations:** 1 School of Medicine, West Virginia University, Morgantown, WV 26505 USA; 2 West Virginia Clinical and Translational Science Institute, Morgantown, WV USA; 3 Department of Behavioral Medicine & Psychiatry, West Virginia University, Morgantown, WV USA; 4 Department of Epidemiology, West Virginia University, Morgantown, WV USA; 5 West Virginia Board of Pharmacy, Charleston, WV USA

**Keywords:** Opioids, Law, Prescription opioids, Opiates, Interrupted time series

## Abstract

**Background:**

The Opioid Reduction Act (SB 273) took effect in West Virginia in June 2018. This legislation limited ongoing chronic opioid prescriptions to 30 days’ supply, and first-time opioid prescriptions to 7 days’ supply for surgeons and 3 days’ for emergency rooms and dentists. The purpose of this study was to determine the effect of this legislation on reducing opioid prescriptions in West Virginia, with the goal of informing future similar policy efforts.

**Methods:**

Data were requested from the state Prescription Drug Monitoring Program (PDMP) including overall number of opioid prescriptions, number of first-time opioid prescriptions, average daily morphine milligram equivalents (MME) and prescription duration (expressed as “days’ supply”) given to adults during the 64 week time periods before and after legislation enactment. Statistical analysis was done utilizing an autoregressive integrated moving average (ARIMA) interrupted time series analysis to assess impact of both legislation announcement and enactment while controlling secular trends and considering autocorrelation trends. Benzodiazepine prescriptions were utilized as a control.

**Results:**

Our analysis demonstrates a significant decrease in overall state opioid prescribing as well as a small change in average daily MME associated with the date of the legislation’s enactment when considering serial correlation in the time series and accounting for pre-intervention trends. There was no such association found with benzodiazepine prescriptions.

**Conclusion:**

Results of the current study suggest that SB 273 was associated with an average 22.1% decrease of overall opioid prescriptions and a small change in average daily MME relative to the date of legislative implementation in West Virginia. There was, however, no association of the legislation on first-time opioid prescriptions or days’ supply of opioid medication, and all variables were trending downward prior to implementation of SB 273. The control demonstrated no relationship to the law.

**Supplementary Information:**

The online version contains supplementary material available at 10.1186/s13011-021-00349-y.

## Introduction

Prescription drug misuse is the administration of a prescription drug in a way not intended by the prescriber [1]. This can include taking someone else’s prescription for an appropriate medical complaint, taking by a different route or higher dose than prescribed, or taking a prescription medication to cause mind-altering affects. The most commonly misused prescription medications are those with mind-altering properties such as anti-anxiety medication, stimulants, hypnotics and opioids [2]. In 2017, an estimated 18 million Americans (6% of people over 12 years of age) misused prescription medications at least once in the past year [3].

Nearly half of participants in a large urban methadone treatment program reported their first contact with opioids was through a doctor’s prescription for medical treatment [4]. According to the 2015 National Survey on Drug Use and Health, the most common reason for the misuse of a prescription pain reliever was in fact, to relieve pain [5]. Additionally, more than half of people who misused prescription pain relievers obtained them from friends and family [5].

Between 1999 and 2017, drug overdoses from prescription opioids rose from 3442 to 17,029 and the deaths from prescription opioids in combination with synthetic opioids has been steadily rising since 2014 [6]. In 2011, the Centers for Disease Control and Prevention (CDC) declared that overdoses from prescription drug abuse had become an “epidemic” and since then prescription drug misuse, including misuse of opioid medications, continues to be a significant public health issue [7].

In an effort to reduce the quantity of available opioid medications, many states impose prescription limits on healthcare providers with the ability to prescribe scheduled drugs, but such laws vary by state [8]. Legislation to limit opioid prescriptions is relatively new. Massachusetts passed the first law in 2016 that set a 7-day supply limit for first-time opioid prescriptions. By 2020, the National Conference of State Legislatures (NCSL) reported 63 bills pending or enacted in 24 states to limit opioid prescribing [9].

According to NCSL, most of this legislation imposes day-limits upon new opioid prescriptions. This is generally 3–14 days, with 7 days being the average. Some states specifically set limits for minors or limit specific dosage (i.e., morphine milligram equivalents; MME). Most states differentiate between acute and chronic pain, and some states have a “professional judgement” clause which allows practitioners to override the restrictions in cases which they feel the prescription limit would be detrimental to patient care [9].

West Virginia has had the highest overdose mortality rate in the U.S. for a decade; in 2018 the rate was 51.5 per 100,000 persons, with the vast majority of deaths involving opioids [10]. During this same time, the national rate was 20.7 per 100,000 persons [10]. Additionally, West Virginia also has one the highest per capita rates of opioid prescriptions. In 2017, health care providers in the US wrote 58.7 opioid prescriptions per 100 persons, while in West Virginia the rate was 81.3 per 100 person [11]. Even at this high per capita rate, prescription opioid dispensing was on the decline in West Virginia, with just over 31 million fewer doses of controlled medications dispensed in 2017 than 2016; of these, approximately half were opioids [12]. In addition, deaths attributed to *prescription* opioids within West Virginia decreased by 20% from 2014 to 2017 [13]. Despite the steady decline in prescription opioids, the rates of prescription-related deaths are still high in comparison to the rest of the nation [13].

**Fig. 1:**
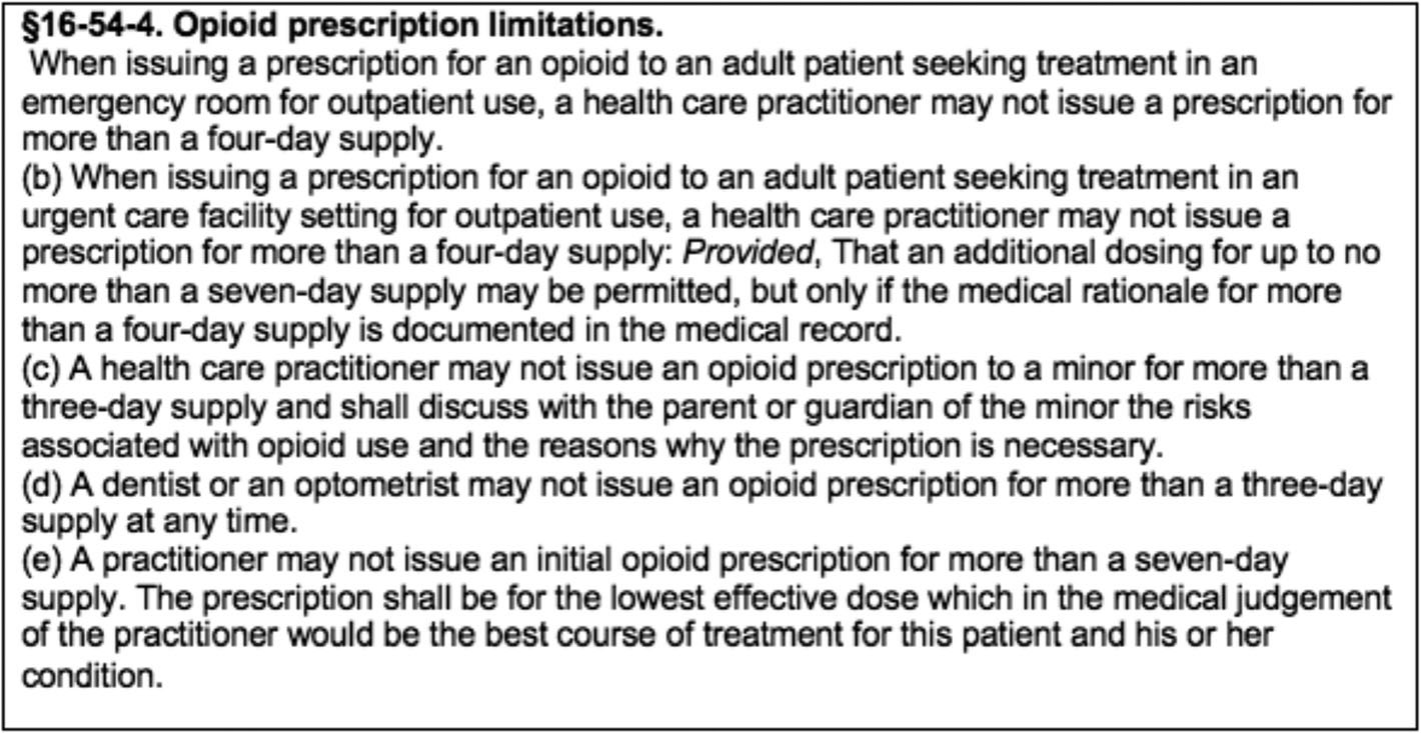
Prescription limitation language in SB 273 (Opioid Reduction Act)

In an effort to reduce the nonmedical use of prescription opioids further, the West Virginia legislature introduced Senate Bill (SB) 273, The Opioid Reduction Act of 2018 (Fig. 1).

On March 27, 2018, the bill was signed and became effective June 7, 2018. This bill establishes prescribing limits for opioid prescriptions by limiting ongoing chronic opioid prescriptions to 30 days’ supply and first-time opioid prescriptions to 7 days’ supply for surgeons and 3 days’ for emergency rooms and dentists, as well as establishing new opioid-related harms counselling and other requirements of prescribers. It does not apply to cancer patients, patients in hospice care, palliative care, residents of long-term care facilities, patients receiving treatment for substance use disorder, and patients receiving on-going opioid treatment as of January 1, 2018 [14].

The purpose of this study is to determine whether SB 273 was associated with a reduction in opioid prescribing in West Virginia, with the goal of informing future policy efforts designed to reduce opioid misuse. To this end, we examined the law’s impact on multiple measures of opioid prescribing including first-time opioid prescription numbers, overall opioid prescription numbers, average day supply, and MME. Utilizing the state PDMP as the information source, we were able to assess the impact of the law across multiple groups of patients including both private and publicly insured patients, and uninsured patients, lending external validity to our results for broad populations of patients. In West Virginia, approximately 28% of patients are covered by Medicaid/CHIP and 24% of patients are Medicare beneficiaries [15, 16]. Seven percent are uninsured [16].

## Materials and methods

### Procedures

We used an interrupted time series quasi-experimental design for state-level data to investigate opioid prescribing practices before and after the bill took effect. This methodology is useful for evaluating effectiveness of health policy changes at a population level [17].

#### Study sample and population

After institutional review board (IRB) approval (Protocol # 1812390727), records from the West Virginia Board of Pharmacy (WVBOP) database were requested. The WVBOP database (PDMP) is an electronic database that stores data on all Schedule II-V controlled substances and opioid antagonists (and other drugs that require identification to purchase, such as pseudoephedrine) that are dispensed by practitioners to West Virginia residents in an outpatient setting by pharmacies, with the exception of correctional facilities, the Indian Health Services, and tribal pharmacies. Additionally, medications dispensed by inpatient hospitals, nursing homes, and MAT (medication assisted treatment) facilities are not collected. Federal military and Veteran’s Affairs facilities are not required to report to the WVBOP. Information regarding diagnosis or reimbursement codes or indications for use are not collected. The data are required to be submitted to the WVBOP every 24 h. RxData Track/CSAPP is the online software, run by Mahantech Corp, used by the WVBOP to track these substances. The data is stored on secure servers and all protected health information (PHI) is kept secure and confidential. The data is accessible by prescribers and dispensers for the purposes of treating their patients. Licensing boards, law enforcement, Office of the Chief Medical Examiner, and other entities have access for investigative purposes only. For this study, all prescriptions dispensed to adult WV patients by WV pharmacies were included. Pediatric prescriptions as well as prescriptions written by veterinarians were excluded. Dental prescriptions were included.

#### Variables assessed

Data requested included the overall number of unique opioid prescriptions, number of unique first-time opioid prescriptions (defined as first opioid prescription for a particular treatment or diagnosis), daily MME and prescription amounts (expressed as “days’ supply” – the terminology in the legislation) given to adults during the time period under analysis. MME was calculated using the standard formula utilized by the CDC: *strength per unit x (number of units/days supply) x MME conversion factor = MME/day*. Because WV has no standardized method to calculate days’ supply, this was done based upon the days’ supply provided by the pharmacist. This reflects the interpretation of dispensers in the real-world system and is reflective of the quantity/instructions or acetaminophen daily limits for combination medications. Prescriptions with missing days’ supply data were excluded from analysis. Data with extreme days’ supply were included. The average days’ supply population consisted of first-time prescriptions during the period under evaluation; similarly, the daily MME was assessed with respect to first-time prescriptions. These variables were selected due to their direct relation to the required components of SB 273.

#### Time period under study

The 54 weeks prior to the announcement of SB 273 (“pre-intervention”) were compared to 10 weeks between announcement and enactment of the law, and the 64 weeks after the enactment of SB 273 (“post-intervention”) in order to provide an adequate number of data points for the ARIMA analysis. We hypothesized that a significant effect would include both a significant level change (immediate change in magnitude after implementation of the law) because of the minimal expected effect lag of a policy change, and a significant slope change (change in the trend before the law as compared to after the law).

#### Control

A similar dataset of benzodiazepine prescriptions were utilized as a control for comparison, as similar societal pressure exists to decrease benzodiazepine prescriptions, but this class of medication was not specifically addressed in SB 273. For example, the CDC guidelines recommend avoidance of benzodiazepines with opioid medications. For this dataset, all benzodiazepines that were dispensed from pharmacies in WV were included. These include alprazolam, chlordiazepoxide, clobazam, clonazepam, clorazepate, diazepam, estazolam, flurazepam, lorazepam, oxazepam, temazepam, and triazolam. Benzodiazepine prescription numbers and days’ supply were calculated using similar methodologies as described above, but diazepam equivalents were not calculated.

### Data analysis

Statistical analysis was done utilizing an autoregressive integrated moving average (ARIMA) interrupted time series analysis (ITS) by a trained statistician. ITS analysis is particularly well equipped to evaluate interventions [18, 19], and the ARIMA model is one of the most common interrupted time series methods [20] and widely used in health care research [17, 21–23]. ARIMA was first introduced by Box and Jenkins in 1976 [24] that combined Auto Regressive (AR) models and Moving Average (MA) models to forecast stationary and non-stationary time series. In AR models, the predicted variable depends linearly on its own, previous values, and an error term. However, in MA models, the predicted variable depends linearly on the current and various past values of white noise or random shock terms. Assuming p is the number of time lags of an AR model and q is the order of an MA model, then an ARIMA process with (*p*, *d*, *q*) order is:

$$ {Y}_t=c+\left({\varphi}_1{\overset{\acute{\mkern6mu}}{Y}}_{t-1}+{\varphi}_2{\overset{\acute{\mkern6mu}}{Y}}_{t-2}+\dots +{\varphi}_p{\overset{\acute{\mkern6mu}}{Y}}_{t-p}\right)-\left({\theta}_1{\varepsilon}_{t-1}+{\theta}_2{\varepsilon}_{t-2}+\dots +{\theta}_q{\varepsilon}_{t-q}\right)+{\varepsilon}_t $$

When *c* is a constant, *X*_*i*_ is the value of time series at time *i*, *φ*_1_, *φ*_2_, …, *φ*_*p*_ are parameters of the model, *ε*_*t*_ is normal random noise at time *t*, *θ*_1_, *θ*_2_, …, *θ*_*q*_ are coefficients of the model, and ${\overset{\acute{\mkern6mu}}{Y}}_t={\nabla}^d{Y}_t$. Here *d* time differencing (∇^*d*^*Y*_*t*_ *or* B^*d*^*Y*_*t*_) helps to produce a stationary process.

For each time series under study, an ARIMA model for the process over the pre-intervention period was first identified (step 1). Then, another ARIMA model with the same orders was fitted to the entire time series to analyze the residuals (step 2). In the final step, an ARIMAX model, the initial ARIMA model with additional regressors or exogenous variables corresponding to announcement and implementation of the legislation, was estimated for the entire time series to identify the intervention effect of both the law announcement and enactment (step 3). This approach has been previously reported [25–27]. In this study, R studio version 1.1.456 based on R version 3.5.1 was used to fit the ARIMA and ARIMAX models.

In the first step, the order of ARIMA was determined with autocorrelation function (ACF) and partial autocorrelation function (PACF). The model was checked for outliers; additive outliers (AO) and innovation outliers (IO) were assessed and added to the model based on the procedure presented by Chang [28]. For testing adequacy, the residuals of the ARIMA models were inspected with ACF, PACF, and Ljung-Box statistics. In case of finding multiple feasible ARIMA models, the model with minimum Akaike’s Information Criterion (AIC) was selected as an appropriate model.

In the last step, a maximum likelihood optimizer was used to estimate the selected ARIMA model with exogenous variables, an ARIMAX model. Exogenous variables in ARIMAX informed the modeling of changes following the interventions. We hypothesized the intervention impact with three functions: step function with immediate effect in mean to detect level change (1 for weeks greater or equal to intervention week; 0 otherwise), ramp function with more gradual effect on the time series to detect slope change (week index after intervention for weeks greater or equal to intervention week; 0 otherwise), and pulse function to capture changes in intervention week (1 for week of intervention; 0 otherwise). Statistical details are presented in Additional file 1.

In order to estimate the impact of the intervention on the response i.e. number of opioid prescriptions, the total number of prescriptions decreased/increased because of the intervention during the post-intervention period (ΔY) was estimated and compared with the total number of prescriptions during that period (Y) by ΔY/*Y*.

Benzodiazepine prescriptions were similarly studied as a control. The detailed modeling is provided in Additional file 1.

## Results

### First-time opioid prescriptions

**Fig. 2:**
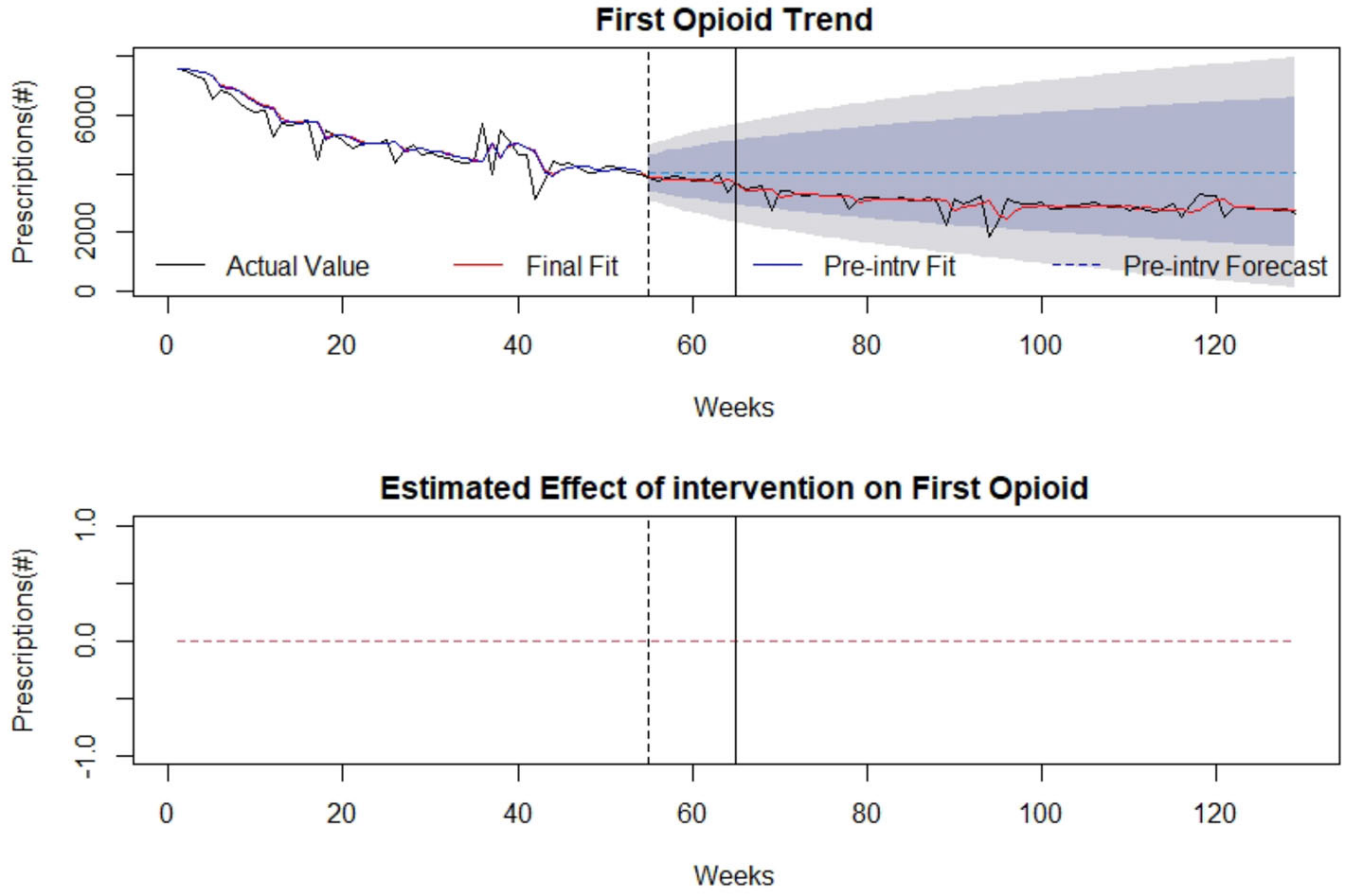
First-Time Opioid Prescriptions: (top) indicates first-time opioid prescriptions in the state of WV over time (in weeks). The broken vertical line indicates legislative announcement and solid vertical line indicates the legislative enactment (intervention). Red dotted line indicates fit of the mathematical model. (bottom) isolates the effect of the intervention

The association of the SB 273 on first-time opioid prescriptions during the time under analysis is demonstrated in Fig. 2 with the timepoints of law announcement and implementation identified by the vertical lines. Overall for the entire time period under study, 509,233 first-time prescriptions for opioid medications were filled. Family Medicine/General Practice (122,838 first-time prescriptions), Dentists/Oral and Maxillofacial Surgery (58,381 first-time prescriptions), and Emergency Medicine (51,060 first-time prescriptions) were the highest prescriber specialties of first-time opioid prescriptions for which data was available. The number of first-time prescriptions overall decreased throughout the pre and post intervention periods, with the initial monthly amount (64 weeks prior to the intervention) being 7563 and the final monthly amount (64 weeks after the intervention) being 2639. There was no significant effect of SB 273 on number of first-time opioid prescriptions after announcing or implementing the legislation based upon this analysis and accounting for pre-intervention trends. Detailed modelling is provided in Additional file 1.

### Overall opioid prescriptions

**Fig. 3:**
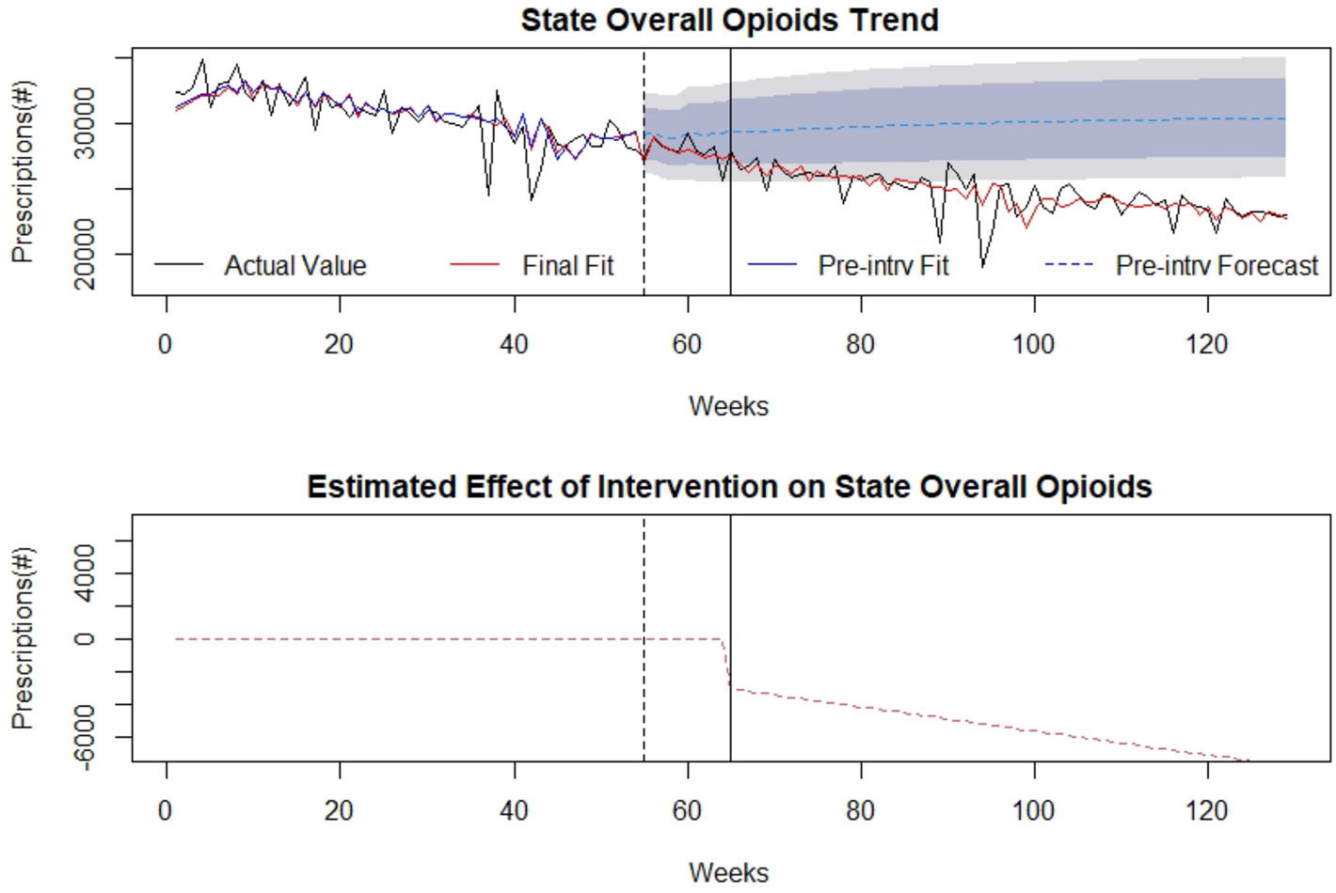
Overall Opioid Prescriptions: (top) indicates overall opioid prescriptions in the state of WV over time (in weeks). The broken vertical line indicates legislative announcement and solid vertical line indicates the legislative enactment (intervention). Red dotted line indicates fit of the mathematical model. (bottom) isolates the effect of the intervention

The effect of the SB 273 on state overall opioid prescriptions during the time under analysis is demonstrated in Fig. 3 with the timepoints of law announcement and implementation identified by the vertical lines. Overall for the entire time period under study, there were 3,523,959 overall opioid prescriptions filled. Family medicine/General Practice (1,285,746 overall prescriptions), Internal Medicine (522,724 overall prescriptions) and Pain Medicine/Pain Management (143,248 overall prescriptions) were the highest prescriber specialties overall for which information was available. The number of overall opioid prescriptions decreased throughout the pre and post-intervention periods, with the initial monthly amount (64 weeks prior to the intervention) being 32,295 prescriptions and the final monthly amount (64 weeks after the intervention) being 22,932 prescriptions. There was a significant level decrease (μ= − 2987 and *p*-value= 0.026) and slope depreciation (μ= − 73.98 and *p*-value= 0.009) in overall opioid prescriptions after implementing the WV legislation based on this analysis. Detailed modelling is provided in Additional file 1. Overall for the entire post-intervention period, it was estimated that there was a 22.1% decrease in the overall number of opioid prescriptions associated with the law implementation. Detailed modelling is provided in Additional file 1.

### Average days’ supply

**Fig. 4:**
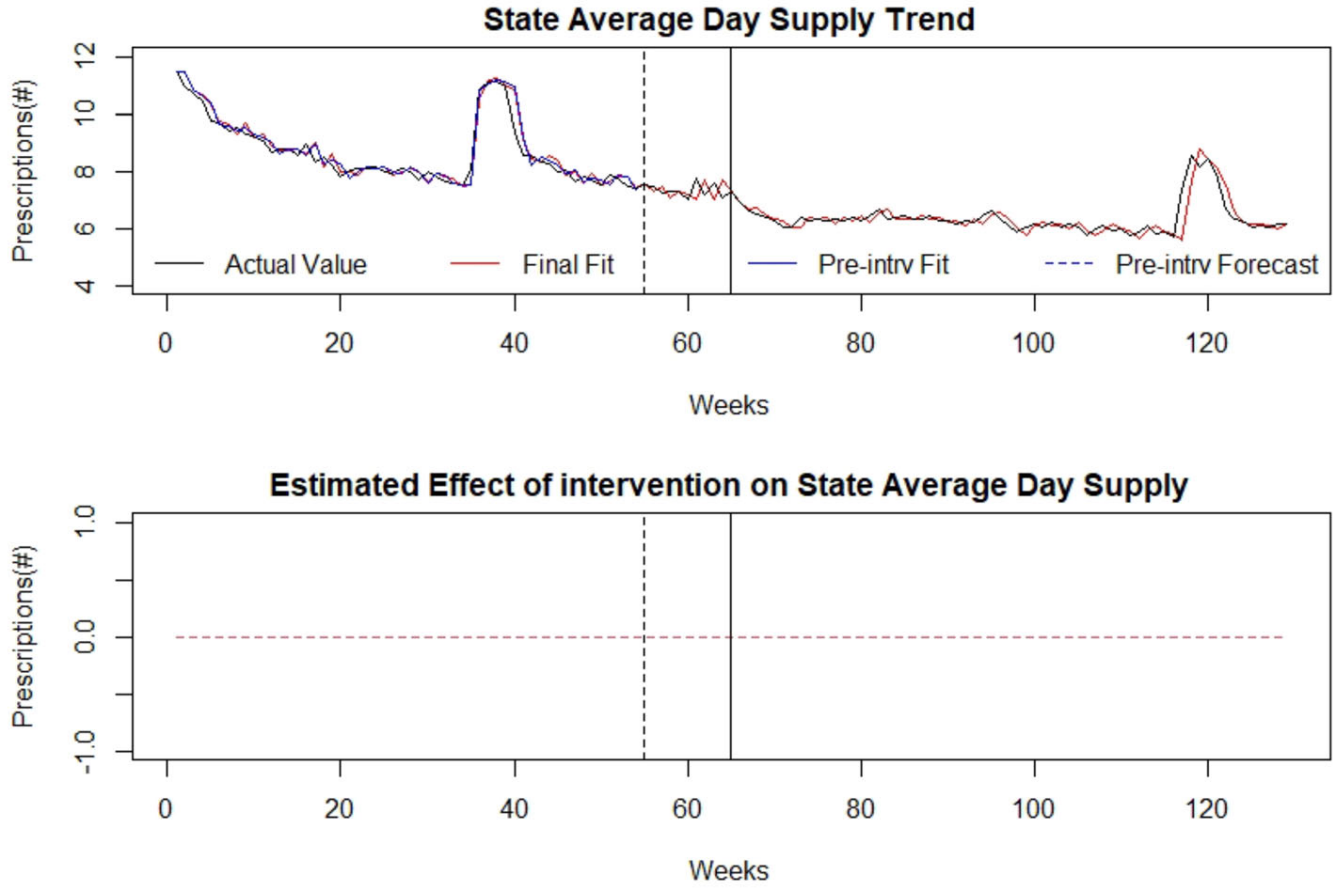
Average Days’ Supply: (top) indicates the average days’ supply of opioid prescriptions in the state of WV over time (in weeks). The broken vertical line indicates legislative announcement and solid vertical line indicates the legislative enactment (intervention). Red dotted line indicates fit of the mathematical model. (bottom) isolates the effect of the intervention

The trend of average days’ supply during the time under analysis is presented in Fig. 4 with the timepoints of law announcement and implementation identified by the vertical lines. Overall the average days’ supply decreased across the pre and post- intervention periods, with the initial average days’ supply being 11.5 days and the final value being 6.2 days. Within the analysis, there was a notable change at timepoint 36 (in both opioid and benzodiazepine series) which will undergo additional study. There was no significant effect of SB 273 on average days’ supply after announcing or implementing the legislation based on our analysis. Detailed modelling is provided in Additional file 1.

### Average daily MME

**Fig. 5:**
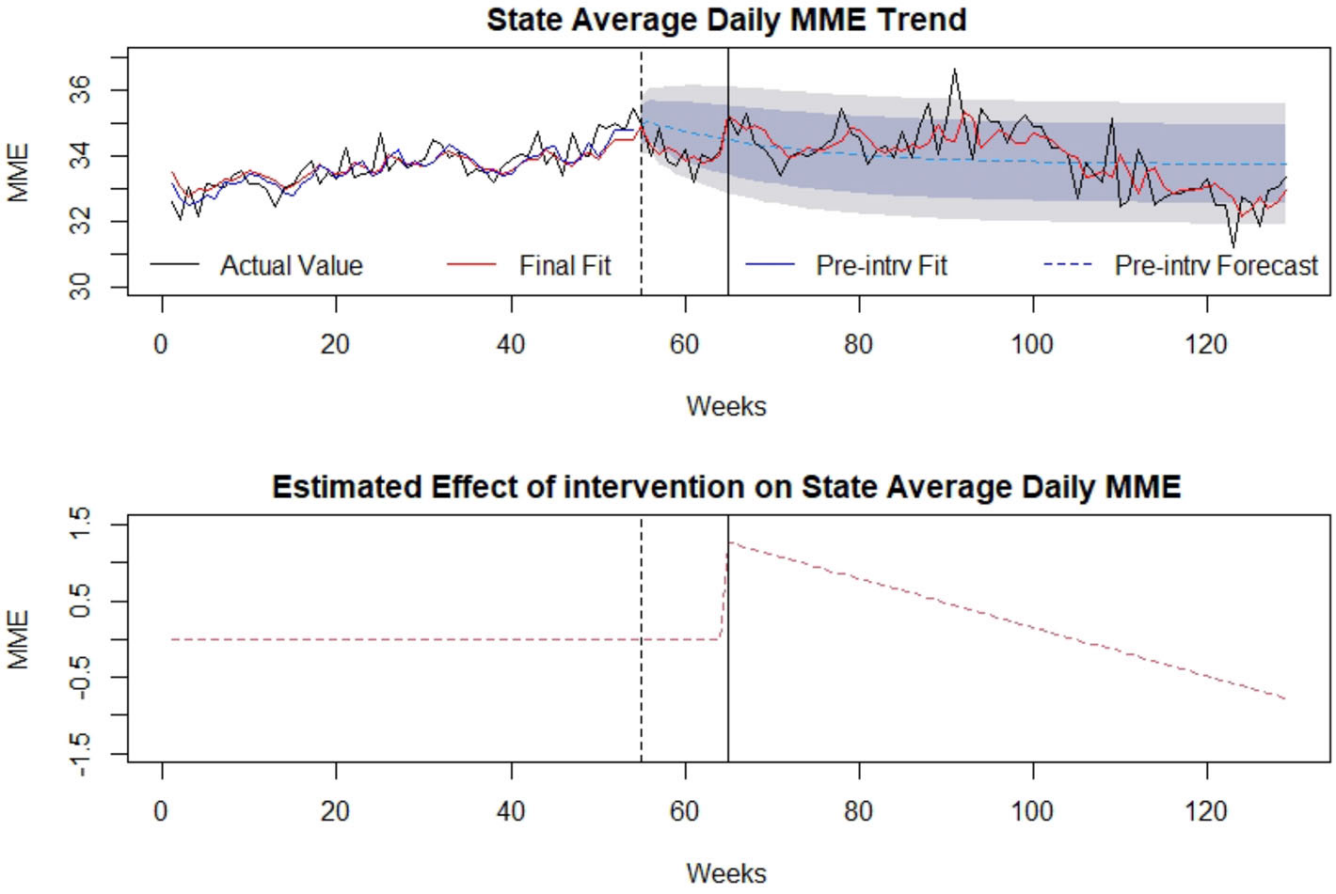
Average Daily Milligram Morphine Equivalents (MME): (top) indicates the average daily MME of opioid prescriptions in the state of WV over time (in weeks). The broken vertical line indicates legislative announcement and solid vertical line indicates the legislative enactment (intervention). Red dotted line indicates fit of the mathematical model. (bottom) isolates the effect of the intervention

The association of the SB 273 on average daily MME during the time under analysis is presented in Fig. 5 with the timepoints of law announcement and implementation identified by the vertical lines. The average daily MME ranged from 31.2 mg to 36.7 mg throughout the time period under study. There was a significant level *increase* (μ= 1.30 and *p*-value= 0.008) and slope *depreciation* (μ= − 0.031 and p-value= 0.003) after implementing the legislation in average daily MME based upon our analysis. Overall, average daily MME suddenly increased, but later followed a decreasing trend and overall was found to decrease after SB 273, however the effect size was small. The law impact on daily MME was estimated as an 0.7% *increase* considering the entire 64 weeks after the law implementation, largely due to the sudden increase in the daily MME immediately after law implementation. If only the final 25 weeks of the study are considered, a 1.1% decrease in daily MME was noted. Detailed modelling is presented in Additional file 1.

### Control

**Fig. 6:**
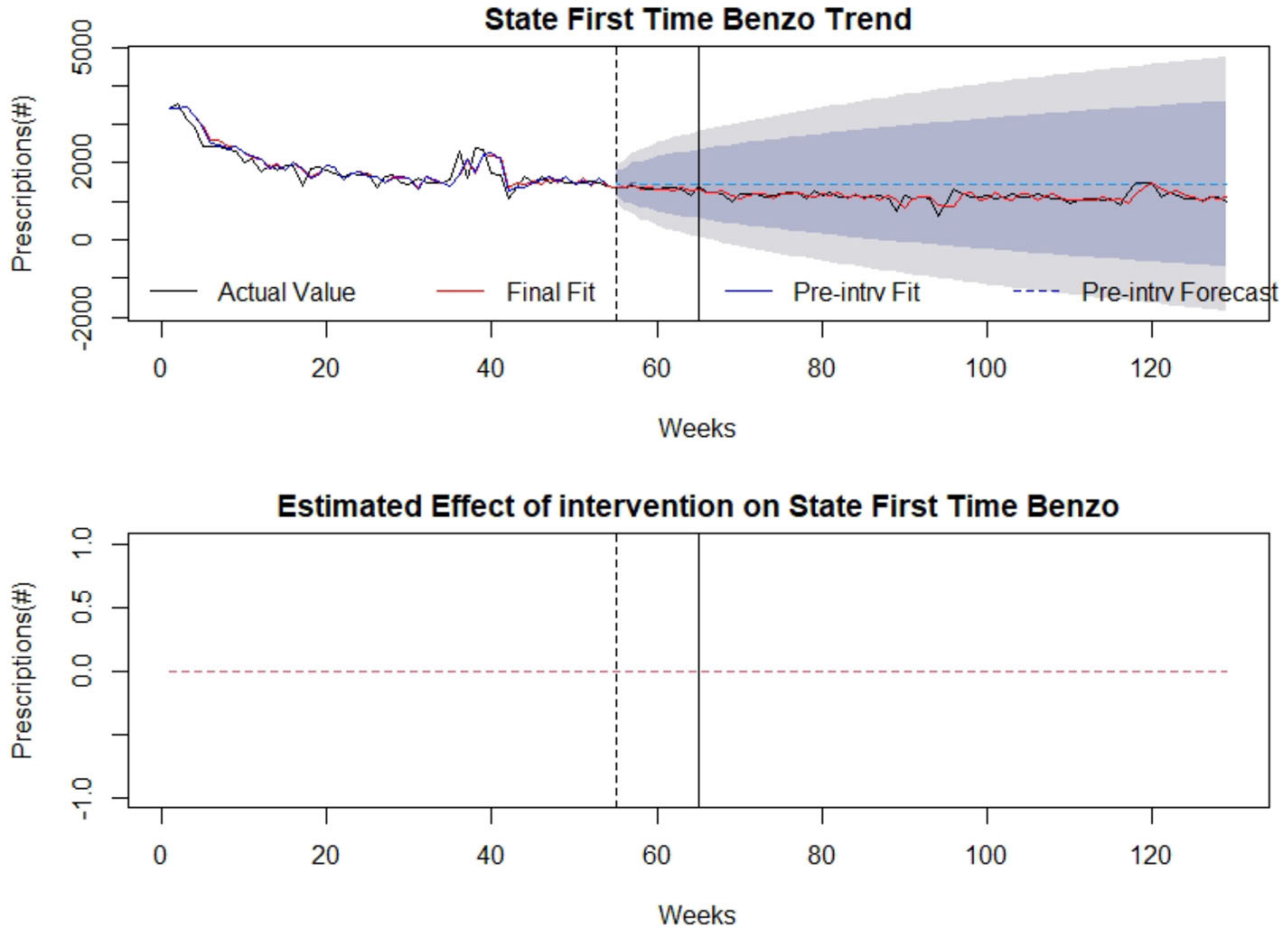
First-time benzodiazepine prescriptions as control: (top) indicates first-time benzodiazepine prescriptions over time (in weeks). The broken vertical line indicates legislative announcement and solid vertical line indicates the legislative enactment (intervention). Red dotted line indicates fit of the mathematical model. (bottom) isolates the effect of the intervention

**Fig. 7:**
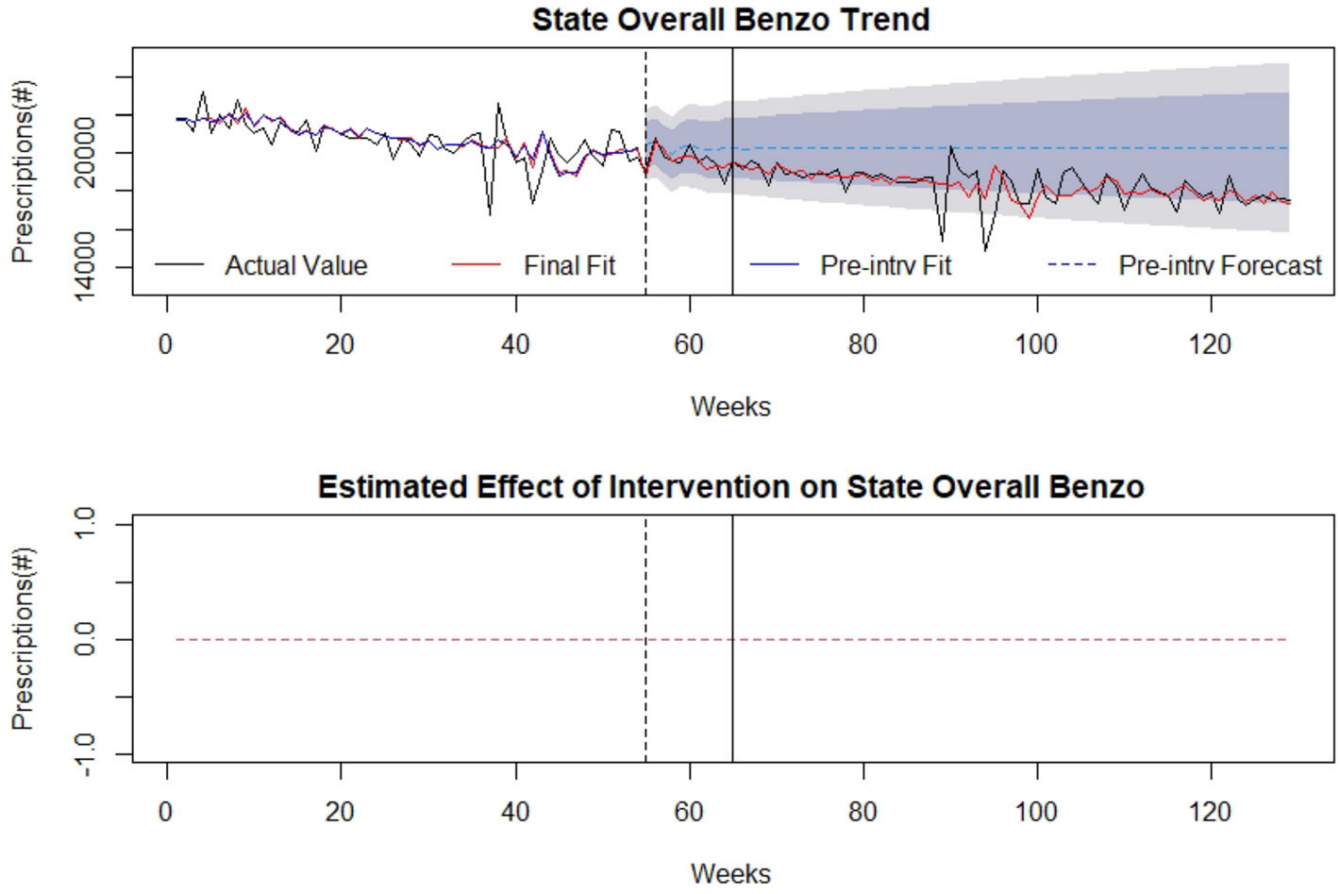
Overall benzodiazepine prescriptions as control: (top) indicates overall benzodiazepine prescriptions over time (in weeks). The broken vertical line indicates legislative announcement and solid vertical line indicates the legislative enactment (intervention). Red dotted line indicates fit of the mathematical model. (bottom) isolates the effect of the intervention

**Fig. 8:**
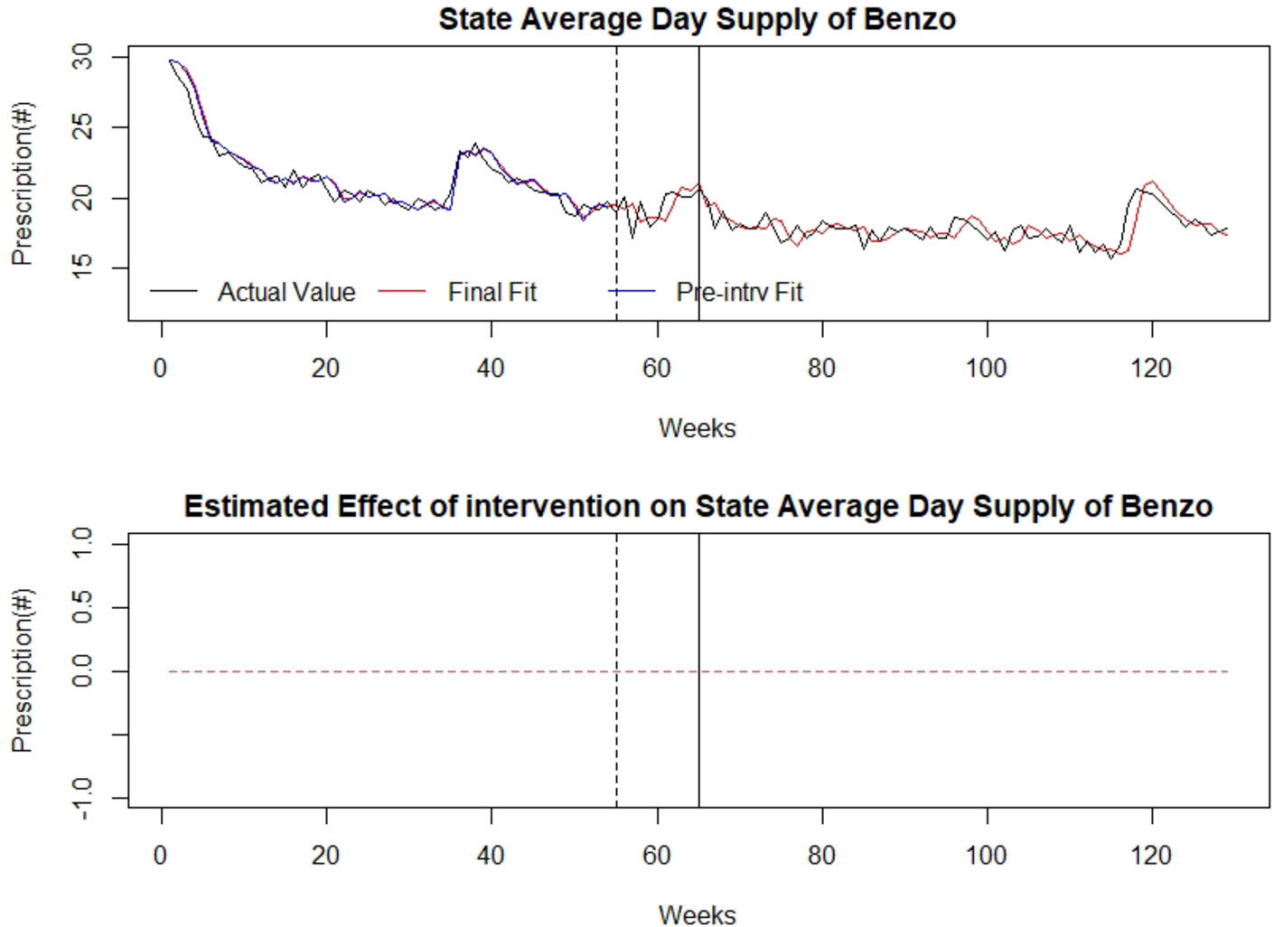
Average Days’ Supply of benzodiazepines as control series: (top) indicates the average days’ supply of benzodiazepine prescriptions over time (in weeks). The broken vertical line indicates legislative announcement and solid vertical line indicates the legislative enactment (intervention). Red dotted line indicates fit of the mathematical model. (bottom) isolates the effect of the intervention

There was no association of the law with overall benzodiazepine prescriptions, first-time benzodiazepine prescriptions, or days’ supply (Figs. 6, 7, 8). Detailed modelling is presented in Additional file 1.

## Discussion

Results of the current study suggest an association between SB 273 and a decrease in overall opioid prescriptions, as well as a small change in average daily MME after legislative implementation in West Virginia. There was, however, no association of the legislation with first-time opioid prescriptions, and all variables were trending downward prior to implementation of SB 273. Furthermore, the daily MME initially *increased* after the law, and the overall decrease, although statistically significant, was minimal in effect size. It is notable that SB 273 specifically does not place a daily average MME limitation on prescribers, although it notes that the “lowest effective dose” should be utilized. In spite of the specific prescription duration limits of SB 273, our analysis indicates that the days’ supply of medication was not significantly affected. Although our study cannot comment on reasons behind a lack of association of the legislation on average days’ supply, we can hypothesize that this finding may have been seen because prescribers were already limiting opioid prescription durations to the limits detailed in the law prior to its enactment. This is suggested because the average days’ supply, although higher at the beginning of the assessment period (13.9 days/prescription in March 2017) had already declined to 7.9 days/prescription prior to signing of the law and continued to decline to 7.3 days/prescription prior to enactment of the law. Chua and colleagues note that opioid prescribing limits may not be effective if the imposed prescribing limits are higher than current clinical practice or patient need [29]. It does appear that current prescribing limits closely mirror current prescribing practices, which may account for the lack of significant quantitative changes in average day supply after the law.

The rationale behind opioid-limiting legislation is that decreasing exposure to opioids amongst opioid naïve patients, as well as decreasing the reservoir of available opioids in the community for misuse, may aid in curtailing the opioid epidemic. This is based upon early findings that 54% of people who misused an opioid obtained it from a friend or relative [30], with the next largest source being directly from prescribers (36%) [30]. Further correlational evidence suggests coincident trends in overdoses with medical prescription of opioids, however it is disputed whether this is a causal relationship and whether previously seen statistics from the “first wave” of the opioid epidemic are still relevant when illicit opioids are currently more prominent sources of adverse events [31]. However, while there is no definitive evidence as to the source of diverted or misused opioids, the study of diversion of medically prescribed opioids is most robust in the acute/first-time opioid prescription phase [30]. In a meta-analysis of multiple studies, Bicket and colleagues found that 67–92% of patients did not use their full opioid prescriptions after surgery, with as few as 9% of patients disposing of them properly [30]. In contrast, given the measures already in place for patients on chronic opioid medications (urine drugs screens, pill counts, etc.) which are not implemented for patients receiving short term opioid prescriptions, it is arguable whether decreasing the ongoing opioid prescription number without decreasing the first-time opioid prescription number (as seen in our study) will have any measurable effect on opioid misuse or diversion. The results of our study do not specifically assess either opioid misuse or diversion, and therefore no conclusions about the effect of this legislation on these metrics can be drawn.

Importantly, the decreased overall prescription number without a corresponding decrease in new opioid prescriptions seem to indicate that patients with chronic pain conditions may be more significantly affected by these legislation effects. This raises concerns of inadequate pain control, or “forced tapering” amongst these patients, particularly given recent concerns that these phenomena may drive illicit use [32]. Confirmation of this finding through clinical data and patient interviews would be helpful to characterize the impact of such legislation on chronic pain patients and any unintended consequences regarding transitions to illicit opioid use.

In contrast to other policy efforts which have undergone study, SB 273 did not have an exception for “professional judgement,” which allows prescribers to override the limits if they feel it is medically required. Agarwal and colleagues [33] have previously postulated that exceptions for “professional judgement” may account for the lack of effect, or minimal effect, of similar laws in other states; concordantly, we found an association in the absence of such an exception within this state specific legislation, which may support that assertion. Greene and colleagues [34] have similarly noted anecdotal evidence that physicians were “getting around” state level prescription limits by writing and back-dating multiple prescriptions, however our data does not support that theory in West Virginia given the continuing decrease in overall number of prescriptions.

Finally, unlike prior studies in other states, we included the law announcement in our analysis in order to capture anticipatory effects on prescription habits. We found no anticipatory effect of the law announcement for any variable. However, our methods do not allow us to discern whether this was due to lack of knowledge/dissemination of the law prior to enactment, or because anticipation of the legislative enactment was not a strong enough driver of prescriber behavior. Accordingly, further study of prescriber-level drivers of prescribing behavior may be warranted.

SB 273 is one of several state-level legislative efforts in recent years to curtail opioid prescribing, with 26 states having enacted these laws by 2017 [35] and 31 states overall having enacted a policy of this type by 2019 [33]. Both Massachusetts and Connecticut instituted 7 day limits for initial opioid prescriptions, with exceptions for chronic, cancer-related, palliative care-related pain similar to the West Virginia legislation; and a “professional judgement” override in contrast to the West Virginia legislation [33]. Florida legislation imposed a more stringent requirement than in other states (3 day limit) [36]. Previous research into the effects of such laws have been mixed. Agarwal and colleagues [33] found variable association of state-level opioid prescribing limits with post-operative opioid prescribing in Massachusetts and Connecticut, but even when a decrease was observed, the magnitude was small. In contrast, study of similar legislation in Rhode Island and Florida demonstrated significant decreases in post-surgical opioid prescribing specifically after state-level opioid prescription limits [36–38].

This conflict in results may be explained by differences in methodology. In Potnuru’s study, pre-intervention indicators of opioid use were compared with post-intervention indicators but there was no accounting for pre-intervention trends, which means the effect reported may have been similar to the downward trend we see in our own data rather than directly attributable to the legislative action [38]. A similar methodology was employed in Reid’s [37] study. In Yenerall and McPheeter’s [39] study, the population under study was a priori defined as “patients currently receiving long-term opioid treatment and most likely to be directly affected by the law” and they limited their analysis to patients who “had at least one prescription with a days’ supply exceeding 30-days in the pre-policy period.” Therefore, since they were assessing the effect of a law that limited the days’ supply of chronic opioids to 30 days, it is unsurprising that they discovered an effect of the legislation in their analysis of this highly selected patient population specifically, which according to their own data is not representative of the majority of prescriptions in the state.

Our study has several additional limitations. Working with the PDMP data in West Virginia we are able to capture prescribing data for prescriptions written and filled within the state, but may have lost data for prescriptions not filled in West Virginia. Several high-volume medical centers are located on state borders, making this limitation potentially relevant. Data is not collected from inpatient hospitals or nursing homes. Furthermore, our study does not include clinical data regarding patient diagnoses, re-admissions due to pain, etc. This is relevant because similar studies have demonstrated opioid-related harms in relation to such legislation. Lastly, while our MME calculation utilizes standard definitions, we utilized day’s supply as reported by dispensers rather than using standardized calculations such as WHO anatomical therapeutic chemical classification defined daily dose, or other standardized methodology which might allow comparison with other datasets.

Further work exploring the specific methods of dissemination and implementation of SB 273 may be relevant to compare West Virginia to other states in which prescribing limits have had varying effects. Similarly, additional assessment of the clinical effect of the overall trends of opioid prescribing in West Virginia are warranted given the decreasing trends seen through the assessment period. While prescribing limits have been attributed by patients and providers as the source of unintended consequences due to decreased prescribing of opioid medications [34], verification of this through rigorous scientific means is warranted.

## Conclusion

Results of the current study suggest an association of SB 273 with a 22.1% decrease in overall opioid prescriptions across 64 weeks after the intervention, as well as a small change in daily average MME associated with the legislation enactment, but no change in first-time opioid prescriptions or days’ supply. There was no change in any metric resulting from announcement of the legislation. Downward trends in first-time opioid prescriptions and average day supply were seen throughout study, but were not associated with SB 273, and further study is indicated to understand drivers behind this trend, as well as unintended consequences. Our data seem to indicate a decrease in ongoing opioid prescriptions rather than new prescriptions, and the effect of this finding on patients with chronic pain conditions is potentially concerning and should be investigated. Finally, it is important to note that the effect of this legislation on diverted or misused opioids was not assessed in this study.

## Supplementary Information

**Additional file 1: Table 1.** Two Sample t-test results ignoring pre-intervention trends and autocorrelations. **Table 2.** Total first opioid pre-intervention model. **Table 3.** Total first opioid model with policy implementation regressors. **Table 4.** Total first opioid model with policy implementation and announcement regressors. **Table 5.** State overall opioids pre-intervention model. **Table 6.** Overall opioids model with policy implementation regressors. **Table 7.** Overall opioids model with policy implementation and announcement regressors. **Table 8.** State average day supply pre-intervention model. **Table 9.** Average day supply model with policy implementation regressors. **Table 10.** Average day supply model with policy implementation and announcement regressors. **Table 11.** Average daily MME pre-intervention model. **Table 12.** Average daily MME model with policy implementation regressor. **Table 13.** Average daily MME model with policy implementation and announcement regressors. **Table 14.** State first time Benzo model with policy implementation and announcement regressors. **Table 15.** State overall Benzo model with policy implementation and announcement regressors. **Table 16.** State average day supply Benzo model with policy implementation and announcement regressors. **Figure 1.** Auto-correlation function, and Ljung-Box statistics of total first opioid pre-intervention model. **Figure 2.** Auto-correlation function, and Ljung-Box statistics of state overall opioids pre-intervention model. **Figure 3.** Auto-correlation function, and Ljung-Box statistics of state average day supply pre-intervention model. **Figure 4.** Auto-correlation function, and Ljung-Box statistics of average daily MME pre-intervention model. **Figure 5.** First time Benzo prescriptions in the state of WV over time. The vertical lines indicate interventions. **Figure 6.** Overall Benzo prescriptions in the state of WV over time. The vertical lines indicate interventions. **Figure 7.** Average days’ supply of Benzo prescriptions in the state of WV over time. The vertical lines indicate interventions.

## Data Availability

Data used for this study can be accessed upon request from the Principal Investigator (Dr. Cara Sedney) at csedney@hsc.wvu.edu

## Abbreviations

ACF: Autocorrelation function
AIC: Akaike’s Information Criterion
AO: Additive outliers
AR: Auto regressive
ARIMA: Autoregressive integrated moving average
ARIMAX: ARIMA model with exogenous variables
CDC: Centers for Disease Control and Prevention
IO: Innovation outliers
IRB: Institutional review board
ITS: Interrupted time series analysis
MA: Moving average
MME: Morphine milligram equivalents
NCSL: National Conference of State Legislatures.
PACF: Partial autocorrelation function
PDMP: Prescription Drug Monitoring Program
PHI: Protected health information
SB 237: The Opioid Reduction Act (Senate Bill 273)
WVBOP: West Virginia Board of Pharmacy
