# Supplementary material for: Assessing the impact of a restrictive opioid prescribing law in West Virginia
Source: Subst Abuse Treat Prev Policy. 2021 Feb 1;16:14. doi: 10.1186/s13011-021-00349-y (PMC7852151; doi:10.1186/s13011-021-00349-y)
Supplement: Supplementary file 1 — Additional file 1: Table 1. Two Sample t-test results ignoring pre-intervention trends and autocorrelations. Table 2. Total first opioid pre-intervention model. Table 3. Total first opioid model with policy implementation regressors. Table 4. Total first opioid model with policy implementation and announcement regressors. Table 5. State overall opioids pre-intervention model. Table 6. Overall opioids model with policy implementation regressors. Table 7. Overall opioids model with policy implementation and announcement regressors. Table 8. State average day supply pre-intervention model. Table 9. Average day supply model with policy implementation regressors. Table 10. Average day supply model with policy implementation and announcement regressors. Table 11. Average daily MME pre-intervention model. Table 12. Average daily MME model with policy implementation regressor. Table 13. Average daily MME model with policy implementation and announcement regressors. Table 14. State first time Benzo model with policy implementation and announcement regressors. Table 15. State overall Benzo model with policy implementation and announcement regressors. Table 16. State average day supply Benzo model with policy implementation and announcement regressors. Figure 1. Auto-correlation function, and Ljung-Box statistics of total first opioid pre-intervention model. Figure 2. Auto-correlation function, and Ljung-Box statistics of state overall opioids pre-intervention model. Figure 3. Auto-correlation function, and Ljung-Box statistics of state average day supply pre-intervention model. Figure 4. Auto-correlation function, and Ljung-Box statistics of average daily MME pre-intervention model. Figure 5. First time Benzo prescriptions in the state of WV over time. The vertical lines indicate interventions. Figure 6. Overall Benzo prescriptions in the state of WV over time. The vertical lines indicate interventions. Figure 7. Average days’ supply of Benzo prescriptions in the state of WV [file 13011_2021_349_MOESM1_ESM.docx]

**Appendix**

**Contents**

[1. T-test (wrong approach) iii](#_Toc49259918)

[2. Autocorrelation Function Plots for Pre-intervention period iii](#_Toc49259919)

[3. Total First Opioid iv](#_Toc49259920)

[3.1. Pre-intervention ARIMA model iv](#_Toc49259921)

[3.2. ARIMAX model for regressor effects iv](#_Toc49259922)

[4. State Overall Opioids vi](#_Toc49259923)

[4.1. Pre-intervention ARIMA model vi](#_Toc49259924)

[4.2. ARIMAX model for regressor effects vi](#_Toc49259925)

[5. State Average Day Supply viii](#_Toc49259926)

[5.1. Pre-intervention ARIMA model viii](#_Toc49259927)

[5.2. ARIMAX model for regressor effects viii](#_Toc49259928)

[6. State Average Daily MME x](#_Toc49259929)

[6.1. Pre-intervention ARIMA model x](#_Toc49259930)

[6.2. ARIMAX model for regressor effects x](#_Toc49259931)

[7. Benzo Control Series xii](#_Toc49259932)

[7.1. State First Time Model xii](#_Toc49259933)

[7.2. State Overall Model xii](#_Toc49259934)

[7.3. State Average Day Supply Model xiii](#_Toc49259935)

**Tables**

[Table 1- Two Sample t-test results ignoring pre-intervention trends and autocorrelations iii](#_Toc49259936)

[Table 2- Total first opioid pre-intervention model iv](#_Toc49259937)

[Table 3- Total first opioid model with policy implementation regressors iv](#_Toc49259938)

[Table 4- Total first opioid model with policy implementation and announcement regressors v](#_Toc49259939)

[Table 5- State overall opioids pre-intervention model vi](#_Toc49259940)

[Table 6- Overall opioids model with policy implementation regressors vi](#_Toc49259941)

[Table 7- Overall opioids model with policy implementation and announcement regressors vii](#_Toc49259942)

[Table 8- State average day supply pre-intervention model viii](#_Toc49259943)

[Table 9- Average day supply model with policy implementation regressors viii](#_Toc49259944)

[Table 10- Average day supply model with policy implementation and announcement regressors ix](#_Toc49259945)

[Table 11- Average daily MME pre-intervention model x](#_Toc49259946)

[Table 12- Average daily MME model with policy implementation regressor x](#_Toc49259947)

[Table 13- Average daily MME model with policy implementation and announcement regressors xi](#_Toc49259948)

[Table 14- State first time Benzo model with policy implementation and announcement regressors xii](#_Toc49259949)

[Table 15- State overall Benzo model with policy implementation and announcement regressors xii](#_Toc49259950)

[Table 16- State average day supply Benzo model with policy implementation and announcement regressors xiii](#_Toc49259951)

**Figures**

[Figure 1- Auto-correlation function, and Ljung-Box statistics of total first opioid pre-intervention model iv](#_Toc49951042)

[Figure 2- Auto-correlation function, and Ljung-Box statistics of state overall opioids pre-intervention model vi](#_Toc49951043)

[Figure 3- Auto-correlation function, and Ljung-Box statistics of state average day supply pre-intervention model viii](#_Toc49951044)

[Figure 4 Auto-correlation function, and Ljung-Box statistics of average daily MME pre-intervention model x](#_Toc49951045)

[Figure 5- First time Benzo prescriptions in the state of WV over time. The vertical lines indicate interventions. xii](#_Toc49951046)

[Figure 6- Overall Benzo prescriptions in the state of WV over time. The vertical lines indicate interventions. xiii](#_Toc49951047)

[Figure 7- Average days’ supply of Benzo prescriptions in the state of WV over time. The vertical lines indicate interventions. xiii](#_Toc49951048)

1. **T-test** **(wrong approach)**

*Table 1- Two Sample t-test results ignoring pre-intervention trends and autocorrelations*

| Time Series | T value | P-value | Conclusion | 95% Lower Bound (before-after) | 95% Upper Bound (before-after) |
| --- | --- | --- | --- | --- | --- |
| First Opioid | 13.895 | < 2e-16 | Reject Null | 1646.226 | 2197.555 |
| Overall State | 16.165 | < 2e-16 | Reject Null | 4927.235 | 6303.234 |
| Day Supply | 13.229 | < 2e-16 | Reject Null | 1.807 | 2.446 |
| Daily MME | -1.205 | 0.230 | Fail to Reject Null | -0.500 | 0.122 |

Alternative hypothesis: before and after intervention difference in means is not equal to 0

1. **Autocorrelation Function Plots for Pre-intervention period**


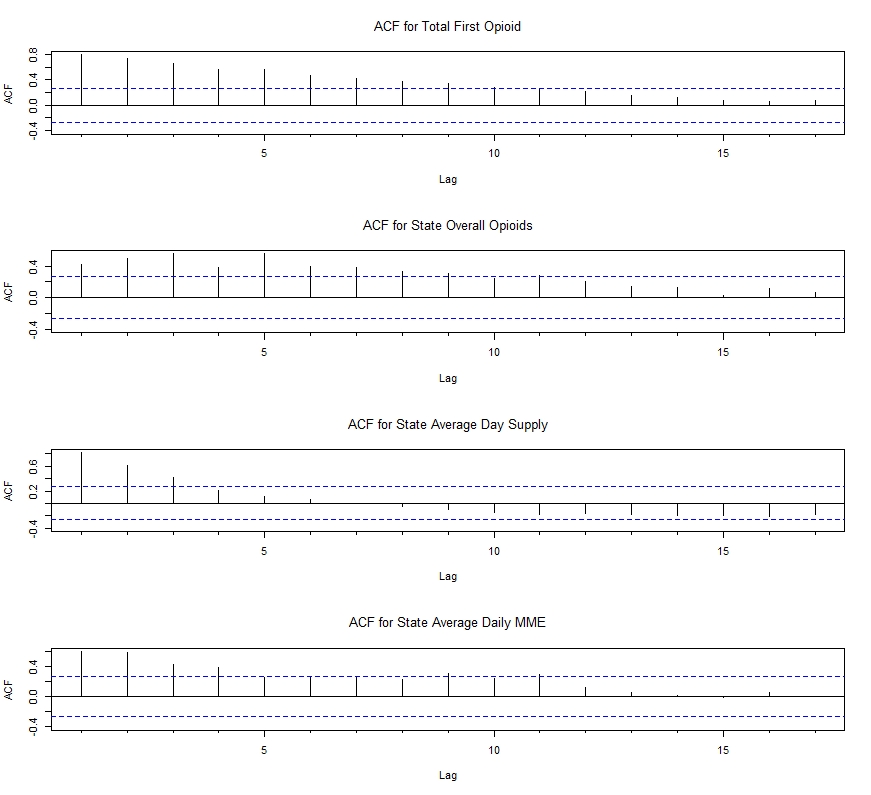


1. **Total First Opioid**
   1. **Pre-intervention ARIMA model**

*Table 2- Total first opioid pre-intervention model*

$$ARIMA\left( 0,1,1 \right)$$

| Coefficients | Estimate | Std. Error | Z value | P-value |
| --- | --- | --- | --- | --- |
| MA(1) | -0.532 | 0.098 | -5.381 | 7e-08 * |

MAPE: 6.35


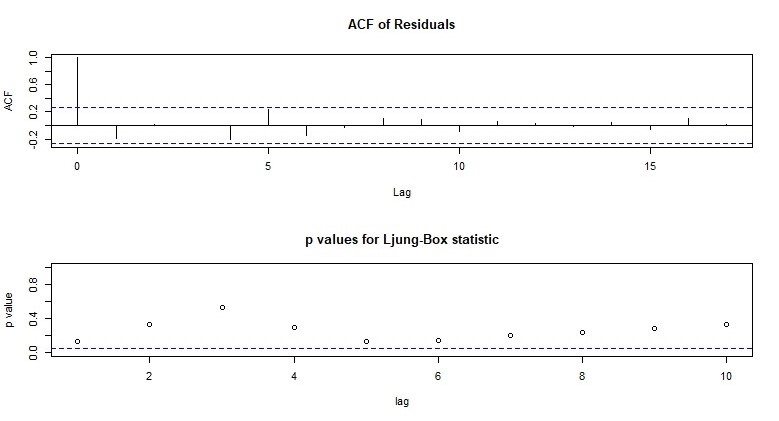


*Figure 1- Auto-correlation function, and Ljung-Box statistics of total first opioid pre-intervention model*

- 1. **ARIMAX model for regressor effects**

*Table 3- Total first opioid model with policy implementation regressors*

$$ARIMA\left( 0,1,1 \right) with pul{se}_{65}, step_{65}, ramp_{65} regressors$$

| Coefficients | Estimate | Std. Error | Z value | P-value |
| --- | --- | --- | --- | --- |
| MA(1) | -0.585 | 0.063 | -9.190 | 2e-16 * |
| Pulse 65 | 229.969 | 370.318 | 0.621 | 0.534 |
| Step 65 | -184.153 | 343.659 | -0.535 | 0.592 |
| Ramp 65 | -11.502 | 19.632 | -0.585 | 0.557 |

*Table 4-* *Total first opioid model with policy implementation and announcement regressors*

$$ARIMA\left( 0,1,1 \right) with pul{se}_{65}, step_{65}, ramp_{65},pul{se}_{55}, step_{55-65}, ramp_{55-65} regressors$$

| Coefficients | Estimate | Std. Error | Z value | P-value |
| --- | --- | --- | --- | --- |
| MA(1) | -0.590 | 0.063 | -9.301 | 2e-16 * |
| Pulse 65 | 230.809 | 369.161 | 0.625 | 0.531 |
| Step 65 | -591.594 | 582.707 | -1.015 | 0.310 |
| Ramp 65 | -11.450 | 19.337 | -0.592 | 0.553 |
| Pulse 55 | -27.063 | 398.880 | -0.067 | 0.945 |
| Step 55-65 | -128.844 | 400.046 | -0.322 | 0.747 |
| Ramp 55-65 | -32.250 | 65.839 | -0.489 | 0.624 |

1. State Overall Opioids
   1. **Pre-intervention ARIMA model**

*Table 5-* *State overall opioids pre-intervention model*

$$ARIMA\left( 5,0,0 \right)$$

| Coefficients | Estimate | Std. Error | Z value | P-value |
| --- | --- | --- | --- | --- |
| AR(1) | 6.829e-03 | 1. 288e-01 | 0.053 | 0.957 |
| AR(2) | 1.302e-01 | 1.271e-01 | 1.024 | 0.305 |
| AR(3) | 3.208e-01 | 1.193e-01 | 2.930 | 0.007 * |
| AR(4) | 1.072e-01 | 1.311e-01 | 0.817 | 0.413 |
| AR(5) | 3.353e-01 | 1.302e-01 | 2.574 | 0.010 * |
| Intercept | 3.0607e+04 | 1.3582e+03 | 22.535 | 2e-16 * |

MAPE: 3.60


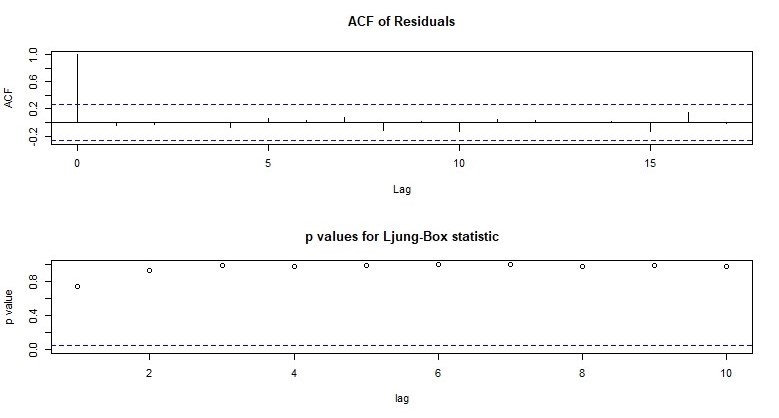


*Figure 2- Auto-correlation function, and Ljung-Box statistics of state overall opioids pre-intervention model*

- 1. **ARIMAX model for regressor effects**

*Table 6- Overall opioids model with policy implementation regressors*

$$ARIMA\left( 5,0,0 \right) with pul{se}_{65}, step_{65}, ramp_{65} regressors$$

| Coefficients | Estimate | Std. Error | Z value | P_value |
| --- | --- | --- | --- | --- |
| AR(1) | 9.111e-02 | 8.049e-02 | 1.132 | 0.257 |
| AR(2) | 5.781e-02 | 7.966e-02 | 0.725 | 0.468 |
| AR(3) | 2.819e-01 | 7.610e-02 | 3.704 | 0.0002 * |
| AR(4) | 8.615e-02 | 8.120e-02 | 1.060 | 0.288 |
| AR(5) | 4.067e-01 | 8.091e-02 | 5.026 | 4.e-07 * |
| Intercept | 2.996e+04 | 1.422e+03 | 21.056 | 2e-16 * |
| Pulse 65 | 2.916e+02 | 1.307e+03 | 0.223 | 0.823 |
| Step 65 | -8.858e+02 | 8.949e+02 | -0.989 | 0.322 |
| Ramp 65 | -8.729e+01 | 3.643e+01 | -2.396 | 0.016 * |

*Table 7- Overall opioids model with policy implementation and announcement regressors*

$$ARIMA\left( 5,0,0 \right) with pul{se}_{65}, step_{65}, ramp_{65},pul{se}_{55}, step_{55-65}, ramp_{55-65} regressors$$

| Coefficients | Estimate | Std. Error | Z value | P_value |
| --- | --- | --- | --- | --- |
| AR(1) | 6.511e-02 | 8.102e-02 | 0.803 | 0.421 |
| AR(2) | 3.031e-02 | 8.041e-02 | 0.376 | 0.706 |
| AR(3) | 2.708e-01 | 7.716e-02 | 3.510 | 0.0004* |
| AR(4) | 8.506e-02 | 8.252e-02 | 1.030 | 0.302 |
| AR(5) | 3.974e-01 | 8.259e-02 | 4.812 | 1e-06 * |
| Intercept | 3.054e+04 | 8.889e+02 | 34.359 | 2e-16 * |
| Pulse 65 | 5.661e+02 | 1.297e+03 | 0.436 | 0.662 |
| Step 65 | -2.987e+03 | 1.345e+03 | -2.221 | 0.026 * |
| Ramp 65 | -7.398e+01 | 2.837e+01 | -2.607 | 0.009 * |
| Pulse 55 | -1.910e+03 | 1.410e+03 | -1.354 | 0.175 |
| Step 55-65 | -5.942e+01 | 1.031e+03 | -0.057 | 0.954 |
| Ramp 55-65 | -2.358e+02 | 1.613e+02 | -1.461 | 0.143 |

1. **State Average Day Supply**
   1. **Pre-intervention ARIMA model**

*Table 8- State average day supply pre-intervention model*

$$ARIMA\left( 0,1,3 \right) with week 36 as outlier$$

| Coefficients | Estimate | Std. Error | Z value | P_value |
| --- | --- | --- | --- | --- |
| MA(1) | 0.136 | 0.138 | 0.98 | 0.327 |
| MA(2) | 0.203 | 0.171 | 1.183 | 0.236 |
| MA(3) | 0.461 | 0.250 | 1.839 | 0.065 * |
| AO (at36) | -0.743 | 0.444 | -1.672 | 0.094 |
| IO (at36) | 3.403 | 0.596 | 5.707 | 1e-08 * |

MAPE= 2.71


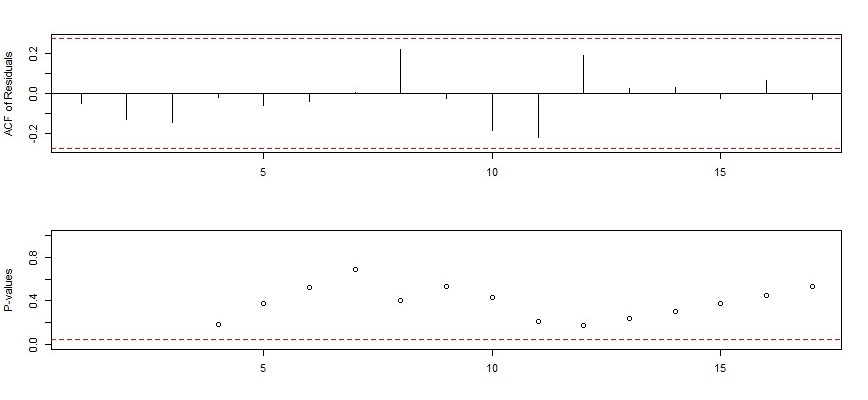


*Figure 3- Auto-correlation function, and Ljung-Box statistics of state average day supply pre-intervention model*

- 1. **ARIMAX model for regressor effects**

*Table 9- Average day supply model with policy implementation regressors*

$$ARIMA\left( 0,1,3 \right) with pul{se}_{65}, step_{65}, ramp_{65} and outlier regressors$$

| Coefficients | Estimate | Std. Error | Z value | P_value |
| --- | --- | --- | --- | --- |
| MA(1) | 0.194 | 0.095 | 2.034 | 0.041 * |
| MA(2) | 0.117 | 0.109 | 1.071 | 0.283 |
| MA(3) | 0.240 | 0.109 | 2.198 | 0.027 * |
| AO (at36) | -0.501 | 0.363 | -1.379 | 0.167 |
| IO (at36) | 3.152 | 0.549 | 5.734 | 9e-09 * |
| Pulse 65 | 0.350 | 0.348 | 1.004 | 0.315 |
| Step 65 | 0.091 | 0.521 | 0.175 | 0.860 |
| Ramp 65 | -0.010 | 0.066 | -0.153 | 0.878 |

*Table 10- Average day supply model with policy implementation and announcement regressors*

$$ARIMA\left( 0,1,3 \right) with pul{se}_{65}, step_{65}, ramp_{65},pul{se}_{55}, step_{55-65}, ramp_{55-65} and outlier regressors$$

| Coefficients | Estimate | Std. Error | Z value | P_value |
| --- | --- | --- | --- | --- |
| MA(1) | 0.199 | 0.095 | 2.082 | 0.037 * |
| MA (2) | 0.124 | 0.109 | 1.136 | 0.255 |
| MA (3) | 0.251 | 0.111 | 2.260 | 0.023 * |
| AO (at36) | -0.505 | 0.363 | -1.391 | 0.164 |
| IO (at36) | 3.147 | 0.549 | 5.724 | 1e-08 * |
| Pulse 65 | 0.373 | 0.350 | 1.066 | 0.286 |
| Step 65 | -0.444 | 1.739 | -0.255 | 0.798 |
| Ramp 65 | -0.007 | 0.067 | -0.116 | 0.907 |
| Pulse 55 | -0.086 | 0.349 | -0.247 | 0.804 |
| Step 55-65 | 0.337 | 0.567 | 0.594 | 0.552 |
| Ramp 55-65 | -0.082 | 0.173 | -0.474 | 0.635 |

1. **State Average Daily MME**
   1. **Pre-intervention ARIMA model**

*Table 11- Average daily MME pre-intervention model*

$$ARIMA\left( 2,0,0 \right)$$

| Coefficients | Estimate | Std. Error | Z value | P_value |
| --- | --- | --- | --- | --- |
| AR(1) | 0.374 | 0.115 | 3.246 | 0.001 * |
| AR(2) | 0.534 | 0.122 | 4.374 | 1e-05 * |
| Intercept | 33.720 | 0.546 | 61.707 | 2e-16 * |

MAPE= 1.12


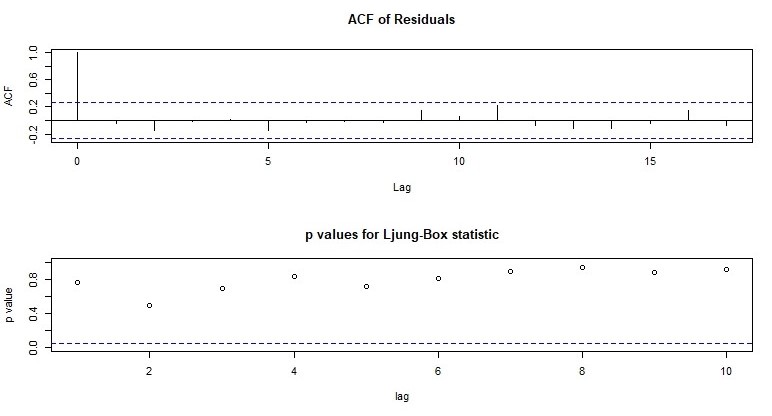


*Figure 4 Auto-correlation function, and Ljung-Box statistics of average daily MME pre-intervention model*

- 1. **ARIMAX model for regressor effects**

*Table 12- Average daily MME model with policy implementation regressor*

$$ARIMA\left( 2,0,0 \right) with pul{se}_{65}, step_{65}, ramp_{65} regressors$$

| Coefficients | Estimate | Std. Error | Z value | P_value |
| --- | --- | --- | --- | --- |
| AR(1) | 0.387 | 0.084 | 4.589 | 4e-06 * |
| AR(2) | 0.276 | 0.086 | 3.202 | 0.001 * |
| Intercept | 33.718 | 0.217 | 154.750 | 2e-16 * |
| Pulse 65 | 0.142 | 0.605 | 0.235 | 0.813 |
| Step 65 | 1.216 | 0.444 | 2.739 | 0.006 * |
| Ramp 65 | -0.030 | 0.010 | -2.843 | 0.004 * |

*Table 13- Average daily MME model with policy implementation and announcement regressors*

$$ARIMA\left( 2,0,0 \right) with pul{se}_{65}, step_{65}, ramp_{65},pul{se}_{55}, step_{55-65}, ramp_{55-65} regressors$$

| Coefficients | Estimate | Std. Error | Z value | P_value |
| --- | --- | --- | --- | --- |
| AR(1) | 0.389 | 0.085 | 4.577 | 4e-06 * |
| AR (2) | 0.277 | 0.087 | 3.177 | 0.001 * |
| Intercept | 33.693 | 0.238 | 141.031 | 2e-16 * |
| Pulse 65 | 0.232 | 0.630 | 0.369 | 0.711 |
| Step 65 | 1.303 | 0.493 | 2.643 | 0.008 * |
| Ramp 65 | -0.031 | 0.011 | -2.880 | 0.003 * |
| Pulse 55 | 0.569 | 0.704 | 0.808 | 0.418 |
| Step 55-65 | -0.459 | 0.797 | -0.576 | 0.564 |
| Ramp 55-65 | 0.079 | 0.111 | 0.717 | 0.473 |

1. **Benzo Control Series**
   1. **State First Time Model**

*Table 14- State first time Benzo model with policy implementation and announcement regressors*

$$ARIMA\left( 0,1,6 \right) with pul{se}_{65}, step_{65}, ramp_{65},pul{se}_{55}, step_{55-65}, ramp_{55-65} regressors$$

| Coefficients | Estimate | Std. Error | Z value | P-value |
| --- | --- | --- | --- | --- |
| MA(1) | -0.233 | 0.085 | -2.719 | 0.006 * |
| MA(2) | -0.047 | 0.092 | -0.508 | 0.611 |
| MA(3) | 0.018 | 0.086 | 0.210 | 0.833 |
| MA(4) | -0.249 | 0.086 | -2.885 | 0.003 * |
| MA(5) | 0.366 | 0.093 | 3.906 | 9.e-05* |
| MA(6) | -0.180 | 0.088 | -2.045 | 0.040 * |
| Pulse 65 | 141.513 | 170.431 | 0.830 | 0.406 |
| Step 65 | -238.1 | 455.863 | -0.522 | 0.601 |
| Ramp 65 | -2.969 | 15.766 | -0.188 | 0.850 |
| Pulse 55 | -81.694 | 192.276 | -0.424 | 0.670 |
| Step 55-65 | -18.463 | 251.748 | -0.073 | 0.941 |
| Ramp_55-65 | -22.014 | 48.373 | -0.455 | 0.649 |


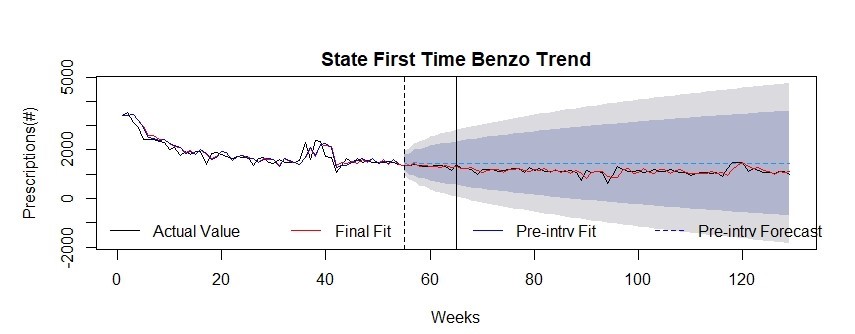


*Figure 5- First time Benzo prescriptions in the state of WV over time. The vertical lines indicate interventions.*

- 1. **State Overall Model**

*Table 15- State overall Benzo model with policy implementation and announcement regressors*

$$ARIMA\left( 4,1,0 \right) with pul{se}_{65}, step_{65}, ramp_{65},pul{se}_{55}, step_{55-65}, ramp_{55-65} regressors$$

| Coefficients | Estimate | Std. Error | Z value | P-value |
| --- | --- | --- | --- | --- |
| AR(1) | -0.965 | 0.078 | -12.222 | 2e-16 * |
| AR(2) | -0.948 | 0.100 | -9.441 | 2e-16 * |
| AR(3) | -0.655 | 0.099 | -6.583 | 4e-11 * |
| AR(4) | -0.445 | 0.078 | -5.652 | 1e-08 * |
| Pulse 65 | -147 | 804 | -0.182 | 0.854 |
| Step 65 | -980 | 838 | -1.169 | 0.242 |
| Ramp 65 | -27.1 | 27.6 | -0.980 | 0.326 |
| Pulse 55 | -1480 | 873 | -1.690 | 0.090 |
| Step 55-65 | 153 | 598 | 0.255 | 0.798 |
| Ramp 55-65 | -126 | 97.3 | -1.298 | 0.194 |


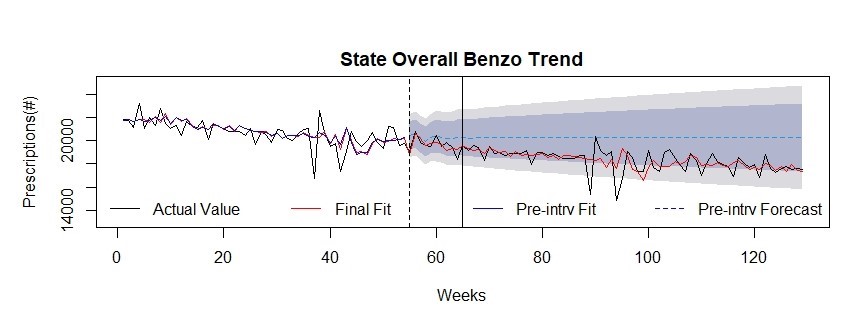


*Figure 6- Overall Benzo prescriptions in the state of WV over time. The vertical lines indicate interventions.*

- 1. **State Average Day Supply Model**

*Table 16- State average day supply Benzo model with policy implementation and announcement regressors*

$$ARIMA\left( 0,1,2 \right) with pul{se}_{65}, step_{65}, ramp_{65},pul{se}_{55}, step_{55-65}, ramp_{55-65} and outlier regressors$$

| Coefficients | Estimate | Std. Error | Z value | P-value |
| --- | --- | --- | --- | --- |
| MA(1) | -0.340 | 0.089 | -3.787 | 0.0001* |
| MA(2) | 0.289 | 0.112 | 2.563 | 0.010 * |
| AO (at36) | 0.159 | 0.814 | 0.195 | 0.844 |
| IO (at36) | 3.003 | 1.001 | 2.998 | 0.002 * |
| Pulse 65 | 1.217 | 0.825 | 1.475 | 0.140 |
| Step 65 | 0.125 | 2.737 | 0.045 | 0.963 |
| Ramp 65 | -0.030 | 0.101 | -0.301 | 0.763 |
| Pulse 55 | 0.098 | 0.871 | 0.112 | 0.910 |
| Step 55-65 | -0.157 | 1.110 | -0.141 | 0.887 |
| Ramp 55-65 | 0.043 | 0.277 | 0.158 | 0.874 |


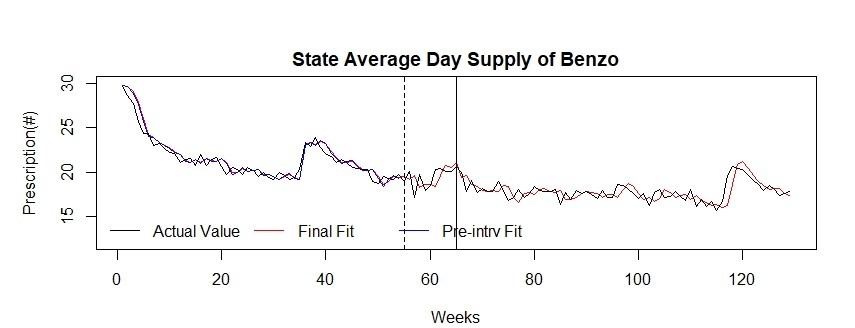


*Figure 7- Average days’ supply of Benzo prescriptions in the state of WV over time. The vertical lines indicate interventions.*
